# Comment on Honndorf et al. Comparative Analytics and Pharmacodynamics of the Complex Protein-Free Botulinum Toxin Type A Formulations DaxibotulinumtoxinA, IncobotulinumtoxinA and RelabotulinumtoxinA. *Toxins* 2026, *18*, 142

**DOI:** 10.3390/toxins18070290

**Published:** 2026-07-03

**Authors:** Ulf Ståhl, Emil Hamnevik, Åsa Liljegren Sundberg, Cecilia Persson, Inna Prygova

**Affiliations:** Galderma, Seminariegatan 21, 752 28 Uppsala, Sweden

In their recent paper comparing three complexing protein-free botulinum toxin A products available on the market [1], Honndorf et al. make several scientifically unfounded statements that in our view could mislead both practitioners and patients.

Firstly, the authors infer that the excipient polysorbate 80 contained in relabotulinumtoxinA (RELA) is toxic for neuronal cells, a conclusion drawn from in vitro cell culture experiments which required significant dilution of the product to avoid cytotoxicity. However, this in vitro-observed toxicity is a well-known artefact when attempting to use a human-formulated product in an in vitro non-dynamic system and not reflective of clinical toxicity. Polysorbate 80 is a very common excipient (Generally Recognized As Safe list/Inactive Ingredient Database/US FDA) administered intramuscularly, subcutaneously, or intravenously in humans, for example in numerous vaccines (Gardasil^®^ 9 [HPV vaccine], as well as in Prevnar 13^®^ [pneumococcal conjugate vaccine]), anticonception (e.g., Depo-Provera [medroxiprogesteronacetate]), dupilumab for atopic dermatitis, or oncology products (e.g., taxotere or generics; antibodies Rituxan^®^ Rituximab) at amounts similar to or higher than those present in RELA. Moreover, in the Phase 3 clinical trial program, single and repeat intramuscular injections of RELA in more than 1700 patients and placebo (containing RELA excipients) in more than 200 patients did not reveal any specific toxicity related to the injection of polysorbate 80 at the concentration used. Hence, the available clinical evidence does not support this conclusion.

Secondly, the authors concluded in their publication that, based on the clinical studies conducted with INCO [2] and RELA [3], the two commercial products have similar efficacy. The authors deliberately referenced the publication from a clinical Phase II study [2] wherein INCO was assessed at doses far higher (50 U, 75 U) than the sole approved dose (20 U) for treating glabellar lines. This selective citation renders their statement factually incorrect. By basing their comparison on such inflated, nonapproved doses, the authors ultimately imply that INCO must be administered far beyond its authorized dose to match the efficacy of RELA. This implication is scientifically untenable, unsafe and clinically misleading.

Further, in the discussion, the authors claim that INCO has the highest specific activity, which they base on the ng protein content in a 100 U-vial. This statement is scientifically incorrect, as the authors themselves acknowledge that product-specific units cannot be compared across different botulinum toxin A-based medicinal products. In addition, there is no statistical analysis supporting such a statement. The data they refer to do not seem to have a statistically significant difference as the standard deviations are overlapping. The lack of support for this claim is further reinforced by the Specific Neurotoxin Potency data in U/ng (usually referred to as specific activity) also showing overlapping standard deviations and an apparent lack of statistically significant difference between INCO and RELA. It is also noted that the total neurotoxin protein data for RELA differs by 15% from earlier measurements [4], 0.46 ng/100 U versus 0.54 ng/100 U, without the authors commenting on the possible reasons for this difference.

The conduct of the digit abduction score (DAS) assay, which is the only in vivo functional assay referenced in this publication, did not include an assessment of RELA, despite the authors stating in the abstract that RELA and INCO were compared in vivo. Furthermore, the comparisons between the botulinum toxin products used in the DAS assay were made at doses inducing significant systemic toxicity (weight loss), which leads us to question the conclusions from the DAS assay.

Lastly, in the context of botulinum toxin, the term “spread” refers to the capacity of the botulinum toxin once injected to extend beyond the intended target muscle. This is an undesirable effect, as it may result in clinically relevant adverse events, as highlighted in the black box warning for Xeomin (INCO). In the experiment described as evaluating spread, the authors used an unvalidated experimental model (ex vivo porcine muscle) and did not study the diffusion or spread of botulinum toxin itself. Instead, they exclusively evaluated the diffusion of formulation excipients (e.g., sodium chloride, polysorbate 80, human serum albumin). Despite this limitation, the authors overinterpreted their findings, first concluding that polysorbate 80 diffuses more than human serum albumin while there is no statistical analysis conducted, and then inappropriately extrapolating this observation to infer that RELA toxin spreads more than other botulinum toxin products. Such an inference is not supported by the experimental design and data.

In conclusion, the Honndorf et al. publication contains several methodological limitations, selective citations, and unsupported interpretations that lead to inaccurate conclusions regarding the properties, efficacy, and safety of RELA in comparison to INCO. Given the clinical importance of these products and their widespread use, it is essential that the scientific record accurately reflects the available evidence to avoid misleading patients and healthcare professionals.
